# The influence of platform switching and platform matching on marginal bone loss in immediately inserted dental implants: a retrospective clinical study

**DOI:** 10.1186/s40729-025-00604-y

**Published:** 2025-03-04

**Authors:** Sameh Attia, Tugce Aykanat, Veronika Chuchmová, Kim Natalie Stolte, Ben Harder, Lucas Schilling, Philipp Streckbein, Hans-Peter Howaldt, Abanoub Riad, Sebastian Böttger

**Affiliations:** 1 Department of Periodontology, Oral Medicine and Oral Surgery, Charité – Universitätsmedizin Berlin, Aßmannshauser Straße 4-6, 14197 Berlin, Germany; 2 Department of Oral and Maxillofacial Surgery, Justus-Liebig-University, Klinikstrasse 33, 3, 35392 Giessen, Germany; 3 Department of Public Health, Faculty of Medicine, Masaryk University, Kamenice 5, Brno, 625 00 Czech Republic; 4 Masaryk Centre for Global Health (MCGH), Department of Public Health, Faculty of Medicine, Masaryk University, Brno, 62500 Czech Republic; 5 Department of Clinical Affairs, BEGO Implant Systems GmbH & Co. KG, Wilhelm- Herbst-Str. 1, 28359 Bremen, Germany

**Keywords:** Platform switching, Platform matching, Immediate implant, Marginal bone loss

## Abstract

**Purpose:**

The aim of this retrospective study was to investigate and compare the effects of platform switching (PS) and platform matching (PM) on marginal bone loss (MBL) and clinical parameters in immediately inserted dental implants.

**Methods:**

Thirty-seven patients were included (PS group: twenty-one patients, PM group: sixteen patients), with follow-up periods ranging from six months to 23 years. MBL was measured using orthopantomograms (OPG), and implant success was evaluated using the Buser, Albrektsson, and Attia criteria. Regression analysis was conducted to assess total bone loss.

**Results:**

The BEGO RI implant system was used in 83.8% of cases. Mesial MBL averaged 0.26 mm in the PS group and 0.75 mm in the PM group, while distal MBL was 0.68 mm for the PS group and 0.53 mm for the PM group. A significant difference was observed in mesial MBL, with the PS group showing less bone loss (*p.* = 0.044). Regression analysis indicated that PM implants were associated with significantly greater mesial bone loss compared to PS implants (*p.* = 0.039). No significant differences in implant success were observed between the PS and PM groups based on the Buser score, Albrektsson criteria, and Attia score.

**Conclusion:**

Both PS and PM implants showed comparable long-term functionality. No significant differences were found in total bone loss between the groups, but PS implants showed significantly lower mesial MBL. While both systems are viable for immediate implantation, PS implants may offer advantages in preserving peri-implant bone. Further prospective studies are needed to validate these findings.

**Graphical abstract:**

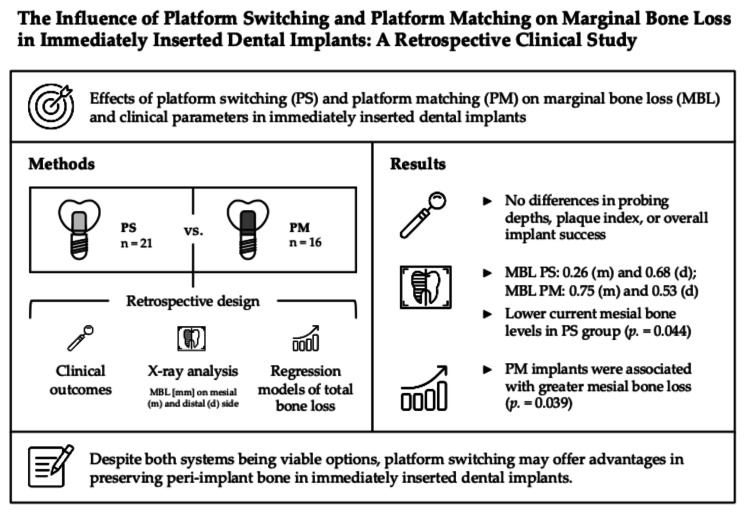

**Supplementary Information:**

The online version contains supplementary material available at 10.1186/s40729-025-00604-y.

## Background

Platform switching (PS), in contrast platform matching (PM), initially referred to a restorative protocol that was firstly reported by Lazzara and Porter as a means of limiting circumferential bone loss around dental implants [1]. The concept of platform switching of dental implants to maintain peri-implant bone levels has become increasingly popular in dental implantology [2–4]. In the 1990s, wide-diameter implants became commercially available. Initially, these implants were restored with standard-diameter abutments instead of wide-diameter abutments due to a lack of matching prosthetic components [4, 5]. Platform switching suggests an abutment or a suprastructure with a diameter at the implant-platform level that is smaller than the implant diameter [1]. This configuration results in a circular horizontal step, which reduces the biologic and mechanical aggressions on the biologic width [6, 7]. One rationale for the beneficial effect of platform switching is to locate the micro-gap of the implant-abutment connection away from the vertical bone-to-implant contact area [7, 8]. Compared with the conventional restorative procedure using an identical implant and suprastructure diameter, PS is suggested to prevent or reduce marginal bone loss (MBL) [1, 3].

Various systematic reviews and meta-analyses have demonstrated a real benefit of PS for patients. Reduced MBL was observed when using PS implant restorations [3, 4, 7, 9, 10]. Twenty-eight publications with a total of 1216 implants with platform switching and 1157 implants with platform matching were included in the meta-analysis by Chrcanovic, Albrektsson and Wennerberg [9]. The results showed less marginal bone loss at implants with platform switching compared to those with platform matching. An increase of the mean difference of MBL between PS and PM groups was observed with the increase in the follow-up time [9]. A recently published systematic review on this topic, including studies with longer follow-up periods between 5 and 10 years, indicated that PS reduced average MBL surrounding implants compared with PM implant-to-abutment connections, favoring the platform-switched approach [4].

Immediate dental implants, which are placed directly after tooth extraction, offer the advantage of reduced treatment time and fewer surgical interventions [11, 12]. However, tooth extraction carries a high risk of bone loss, particularly in the early stages of healing [12]. Studies such as those by Botticelli et al. (2004) and Araújo & Lindhe (2005) have shown significant remodeling of the alveolar ridge post-extraction, leading to both vertical and horizontal bone loss [13, 14]. Therefore, it may be advantageous to use platform switching implants in immediate implantation to prevent potential bone loss. This topic is not well-addressed in the current literature, which is why this study was conceived. The aim is to explore the efficacy of platform switching implants in mitigating bone resorption commonly associated with immediate dental implants, thereby contributing valuable insights to the field and potentially improving clinical outcomes.

The goal of the retrospective study is to investigate and compare platform switching and platform matching concerning the extent of marginal bone loss and further clinical signs of periodontal status in immediately inserted dental implants.

## Materials and methods

### Study design and patient population

In this clinical retrospective study, patients who were treated with endosseous immediate dental implants after tooth extraction at the Clinic for Oral and Maxillofacial Surgery of the University Hospital Gießen, Germany, between the years 2000 and 2023 were included. The patient cohort was assembled after reviewing the surgical plans. The implants must have been under functional load for at least six months. The follow-up examinations took place between June 2023 and February 2024.

### Study parameters

#### Vertical bone loss

The main study parameter is the marginal bone loss measered at the mesial and the distal sides of the implant in both the PS and PM groups. Radiological bone loss was measured using orthopantomograms (OPG). The measurements were performed on a diagnostic monitor using the SIDEXIS XS / 4 Viewer^®^ (Dentsply Sirona, York, USA) software. First, the presence of peri-implant radiolucency was evaluated and accordingly marked. Subsequently, the calculation of crestal, vertical bone loss around the implant was performed. For this purpose, two radiographs taken at different times were compared. Specifically, the crestal bone level on a postoperative OPG was compared with a current OPG taken at the time of examination. The resultant longitudinal vertical bone loss after implantation was calculated by determining the difference between the current and postoperative crestal bone levels. The measurement described above was performed both mesially and distally on the implant.

#### Implant success

In this study, implant success was evaluated according to the criteria established by Buser and Albrektsson, as well as a newly developed criteria by Attia. Buser et al. defined the following criteria in 1990 that a successful implant should meet:

- No persistent pain, foreign body sensation, or dysesthesia.
- No recurrent purulent peri-implant infection anamnestically.
- No mobility of the implant.
- No continuous peri-implant radiolucency.
- Possibility of prosthetic restoration (superstructure).

An implant was considered a failure if any criterion is not met. Explantation corresponded to a failure.

In addition to the success criteria defined by Buser, the criteria established by Albrektsson were also applied. These criteria were:

- The implant should be clinically stable.
- No continuous peri-implant radiolucency should be visible.
- After the first year under load, vertical bone loss should be less than 0.2 mm/year.
- Absence of signs of infection, pain, paresthesia, or injury to the mandibular canal.
- At a five-year observation period, the success rate should be 85%. At the end of a ten-year observation period, the success rate should be 80%.

Only if all criteria were met an implant was considered successful. Here, too, explantation was considered a failure.

Finally, success was evaluated according to the newly developed success score by Attia. This score is divided into four groups:

1. **Knockout Criteria**: If any of these criteria are met, the implant is defined as unsuccessful and is not subject to further investigation. The presence of these criteria precludes later prosthetic restoration.
  Implant mobility.
  Implant fracture.
  Wrong implant position.

2. **Implant-Related Parameters**: Each point can receive up to two points in the evaluation.
  Absence of pain on percussion and palpation.
  Compared to the time of implantation, the annual peri-implant marginal bone resorption should not exceed the calculated value (“y = 1.5 + 0.2 mm * (x-1); y = allowable bone loss, x = age of the implant in years”).
  The average probing depth from four sites should be *≤ 4*mm.
  Absence of pus on probing.
  Absence of bleeding on probing.

3. **Peri-Implant Soft Tissue and Prosthetic Restoration**: Each of these points can receive a maximum of one point.
  Absence of plaque.
  Absence of complications of prosthetic restoration, such as fracture or debonding.
  Presence of healthy mucosa.

4. **Patient Satisfaction**: Each aspect can receive a maximum of one point.
  Absence of foreign body sensation.
  Absence of paresthesia.
  Aesthetic satisfaction.
  Satisfaction with masticatory function.
  Satisfaction with speech ability.

A maximum score of 18 can be achieved, with a score of 0 indicating failure. Implants with a score of 1–6 are rated as satisfactory, 7–12 as good, and 13–18 as very good.

### Inclusion and exclusion criteria for study subjects

The inclusion criteria initially encompassed all patients who received an immediate implantat after extraction. The implant must have been under functional load for at least six months. During the study period, the following exclusion criteria must not apply to the participating subjects: Must not be undergoing radiation therapy in the head-neck area. Must not be receiving treatment with bisphosphonates. Must not be pregnant.

### Statistical analyses

All statistical tests were carried out using SPSS version 28 (IBM Corp., Armonk, NY, USA) and the R-based software Jamovi version 2.3 (The Jamovi Project, Sydney, Australia). Initially, descriptive statistics using means (µ), standard deviations (SD), frequencies (n), and percentages (%) were used to summarize the demographic and clinical characteristics of the study participants. Then inferential statistics were employed to explore the associations between implant-abutment configuration and patients’ characteristics and clinical outcomes. Chi-squared test (χ^2^), Fisher’s exact test, Mann-Whitney test (U), and Spearman’s correlation were utilised. To identify predictors of total bone loss (TBL) at mesial and distal sides, multiple linear regression (MLR) was conducted, adjusting for potential confounders. The statistical significance level was set at *p.*. < 0.05 for all inferential tests.

### Ethics statement/confirmation of patients’ permission

The Ethics committee of the faculty of medicine of Justus-Liebig University Giessen approved the study with the confirmation numbers (126/22). Patients’ consent was obtained from every included patient in the study.

## Results

### Sample characteristics

Out of the 37 included patients, 21 (57%) received platform switching implants, while 16 (43%) received platform matching implants (Table 1). The majority were male (64.9%), with no significant (*p.*. = 0.666) difference between platform switching (61.9%) and platform matching (68.8%) groups. The patients had a mean age at the time of surgery of 46.39 ± 20.86, with no significant difference between the two study groups (*p.*. = 0.175).

**Table 1:** Characteristics of immediate implants inserted at University Hospital of Giessen and Marburg (UKGM) between 2002 and 2023, stratified by implant-abutment configuration (*n* = 37)

Variable	Outcome	Platform-switching (*n* = 21)	Platform-matching (*n* = 16)	Total (*n* = 37)	*p.*
**Sex**	Female	8 (38.1%)	5 (31.3%)	13 (35.1%)	0.666
	Male	13 (61.9%)	11 (68.8%)	24 (64.9%)	
**Age at Operation**	Mean ± SD	50.03 ± 20.66	41.62 ± 20.80	46.39 ± 20.86	0.175
**Chronic Illness**	No	11 (55%)	8 (50%)	19 (52.8%)	0.765
	Yes	9 (45%)	8 (50%)	17 (47.2%)	
**Medications**	No	15 (71.4%)	7 (43.8%)	22 (59.5%)	0.089
	Yes	6 (28.6%)	9 (56.3%)	15 (40.5%)	
**Smoking Status**	No	12 (57.1%)	16 (100%)	28 (75.7%)	**0.005**
	Yes	9 (42.9%)	0 (0%)	9 (24.3%)	
**Toothbrushing**	No	0 (0%)	1 (6.3%)	1 (2.7%)	0.712
	Yes, twice a day	20 (95.2%)	14 (87.5%)	34 (91.9%)	
	Yes, once a day	1 (4.8%)	1 (6.3%)	2 (5.4%)	
**Jaw**	Upper	19 (90.5%)	15 (93.8%)	34 (91.9%)	1.000
	Lower	2 (9.5%)	1 (6.3%)	3 (8.1%)	
**Indication**	Trauma	20 (95.2%)	13 (81.3%)	33 (89.2%)	0.117
	Prior implant explanation	0 (0%)	2 (12.5%)	2 (5.4%)	
	Hypodontia	0 (0%)	1 (6.3%)	1 (2.7%)	
	Root remnant	1 (4.8%)	0 (0%)	1 (2.7%)	
**System**	BEGO	17 (81%)	14 (87.5%)	31 (83.8%)	0.247
	XIVE	4 (19%)	1 (6.3%)	5 (13.5%)	
	Straumann	0 (0%)	1 (6.3%)	1 (2.7%)	
**Implant Diameter**	3.75 *mm*	4 (19%)	1 (6.3%)	5 (13.5%)	0.519
	4.10 *mm*	5 (23.8%)	6 (37.5%)	11 (29.7%)	
	4.50 *mm*	12 (57.1%)	9 (56.3%)	21 (56.8%)	
**Implant Length**	10 *mm*	0 (0%)	1 (6.3%)	1 (2.7%)	**0.006**
	11.50 *mm*	5 (23.8%)	1 (6.3%)	6 (16.2%)	
	12 *mm*	0 (0%)	1 (6.3%)	1 (2.7%)	
	13 *mm*	12 (57.1%)	3 (18.8%)	15 (40.5%)	
	15 *mm*	4 (19%)	10 (62.5%)	14 (37.8%)	
**Service Time**	Mean ± SD	3.18 ± 1.69	9.69 ± 4.09	5.99 ± 4.39	**< 0.001**
**Superstructure**	VMK Single Crown	18 (85.7%)	15 (93.8%)	33 (89.2%)	1.000
	Bridge	1 (4.8%)	0 (0%)	1 (2.7%)	
	Overdenture	2 (9.5%)	1 (6.3%)	3 (8.1%)	

Chi-squared (*χ*^*2*^) test, Fisher’s exact test, and Mann-Whitney (*U*) test was used with a significance level (*p*.) < 0.05

Chronic illnesses and medication usage were present in 47.2% and 40.5% of the patients, respectively, with no significant differences between the study groups (*p.*. = 0.765 and *p.*. = 0.089). Smoking status differed significantly; none of the platform matching patients were smokers, compared to 42.9% in the platform switching group (*p.*. = 0.005). Most patients (91.9%) brushed their teeth twice daily, with no significant difference between the groups (*p.*. = 0.712).

Implants were predominantly placed in the upper jaw (91.9%) in both groups (*p.*. = 1.000).

Trauma was the main indication for implant placement (89.2%), with no significant difference between groups (*p.*. = 0.117), followed by prior implant explanation (5.4%), hypodontia in permanent dentition and extraction of deciduous tooth (2.7%) and root remnant (2.7%).

BEGO RI was the most frequently used implant system (83.8%), with no significant differences between the groups (*p* = 0.247). The most common implant diameter was 4.50 mm (56.8%), and the lengths 13 mm (40.5%) and 15 mm (37.8%) were prevalent. A significantly higher percentage of 15 mm diameter implants was observed in the platform matching group (62.5%) compared to the platform switching group (19%) (*p* = 0.006). Service time was significantly longer for platform matching implants (9.69 ± 4.09 years) versus platform switching implants (3.18 ± 1.69 years) (*p.*. < 0.001). Most patients received a VMK single crown (89.2%), with no significant difference between the groups (*p.*. = 1.000).

### Clinical outcomes

The plaque index showed no significant difference between the platform switching and platform matching groups (*p.* = 0.087), with the majority of patients having a plaque index of 0 (59.5%) (Table 2). Mesial probing depths were comparable across both configurations, with a mean of 2.81 mm (*p.* = 0.440). Similarly, distal probing depths showed no significant difference between the groups, with a mean depth of 2.64 mm (*p.* = 0.937). Vestibular and lingual probing depths were also similar, with means of 2.03 mm and 2.20 mm, respectively, and no significant differences observed (*p.* = 0.814 and *p.* = 0.367).

**Table 2:** Clinical outcomes of immediate implants inserted at University Hospital of Giessen and Marburg (UKGM) between 2002 and 2023, stratified by implant-abutment configuration (*n* = 37)

Variable	Outcome	Platform-switching (*n* = 21)	Platform-matching (*n* = 16)	Total (*n* = 37)	*p*.
**Plaque Index**	0	12 (57.1%)	10 (62.5%)	22 (59.5%)	0.087
	1	4 (19%)	6 (37.5%)	10 (27%)	
	2	5 (23.8%)	0 (0%)	5 (13.5%)	
**Mesial Probing**	1 *mm*	1 (5%)	0 (0%)	1 (2.8%)	0.751
	2 *mm*	4 (20%)	6 (37.5%)	10 (27.8%)	
	3 *mm*	12 (60%)	9 (56.3%)	21 (58.3%)	
	4 *mm*	2 (10%)	1 (6.3%)	3 (8.3%)	
	5 *mm*	1 (5%)	0 (0%)	1 (2.8%)	
	Mean ± SD	2.90 ± 0.85	2.69 ± 0.60	2.81 ± 0.75	0.440
**Distal Probing**	1 *mm*	3 (15%)	0 (0%)	3 (8.3%)	0.409
	2 *mm*	5 (25%)	7 (43.8%)	12 (33.3%)	
	3 *mm*	9 (45%)	7 (43.8%)	16 (44.4%)	
	4 *mm*	3 (15%)	2 (12.5%)	5 (13.9%)	
	Mean ± SD	2.60 ± 0.94	2.69 ± 0.70	2.64 ± 0.83	0.937
**Vestibular Probing**	1 *mm*	3 (15%)	1 (6.3%)	4 (11.1%)	0.392
	2 *mm*	13 (65%)	14 (87.5%)	27 (75%)	
	3 *mm*	4 (20%)	1 (6.3%)	5 (13.9%)	
	Mean ± SD	2.05 ± 0.61	2.00 ± 0.37	2.03 ± 0.51	0.814
**Lingual Probing**	1 *mm*	2 (10.5%)	2 (12.5%)	4 (11.4%)	0.825
	2 *mm*	10 (52.6%)	11 (68.8%)	21 (60%)	
	3 *mm*	6 (31.6%)	3 (18.8%)	9 (25.7%)	
	4 *mm*	1 (5.3%)	0 (0%)	1 (2.9%)	
	Mean ± SD	2.32 ± 0.75	2.06 ± 0.57	2.20 ± 0.68	0.367
**Complaint**	No	15 (71.4%)	11 (68.8%)	26 (70.3%)	1.000
	Yes	6 (28.6%)	5 (31.3%)	11 (29.7%)	
**Overall Satisfaction**	Very Good	19 (90.5%)	13 (81.3%)	32 (86.5%)	0.634
	Good	2 (9.5%)	3 (18.8%)	5 (13.5%)	
**Chewing**	Very Good	17 (81%)	13 (81.3%)	30 (81.1%)	1.000
	Good	3 (14.3%)	3 (18.8%)	6 (16.2%)	
	Satisfactory	1 (4.8%)	0 (0%)	1 (2.7%)	
**Speech**	Very Good	18 (85.7%)	12 (75%)	30 (81.1%)	0.224
	Good	3 (14.3%)	1 (6.3%)	4 (10.8%)	
	Satisfactory	0 (0%)	1 (6.3%)	1 (2.7%)	
	Sufficient	0 (0%)	2 (12.5%)	2 (5.4%)	
**Buser Score**	Success	21 (100%)	15 (93.8%)	36 (97.3%)	0.432
	Failure	0 (0%)	1 (6.3%)	1 (2.7%)	
**Albrektsson Score**	Success	8 (38.1%)	9 (56.3%)	17 (45.9%)	0.272
	Failure	13 (61.9%)	7 (43.8%)	20 (54.1%)	
**Attia Score**	Mean ± SD	16.24 ± 1.04	13.71 ± 6.59	15.11 ± 4.58	0.794
**Pink Score (Photograph)**	Mean ± SD	11.37 ± 3.08	11.13 ± 2.53	11.26 ± 2.81	0.811
**Pink Score (Radiograph)**	Mean ± SD	11.79 ± 1.55	11.80 ± 1.37	11.79 ± 1.45	1.000

Chi-squared (*χ*^*2*^) test, Fisher’s exact test, and Mann-Whitney (*U*) test was used with a significance level (*p*.) < 0.05

Patient complaints and overall satisfaction did not differ significantly between the groups. The majority of patients reported no complaints (70.3%) and very good overall satisfaction (86.5%). Chewing outcomes were highly rated, with 81.1% of patients in both groups reporting very good function (*p.* = 1.000). Speech outcomes were similarly positive, with 81.1% of patients reporting very good results, and no significant difference between the groups (*p.* = 0.224).

**Fig. 1:**
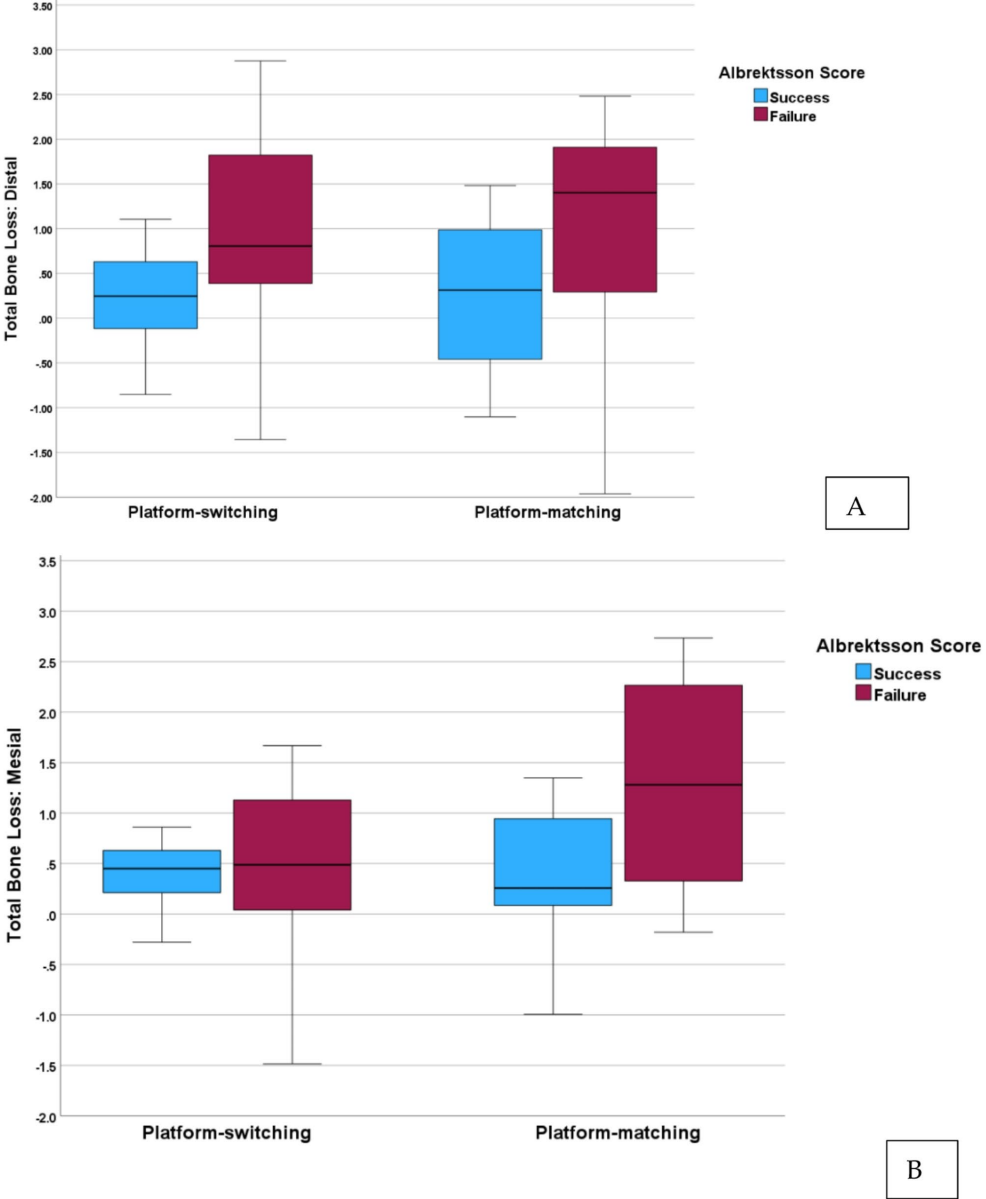
Boxplot of mesial **(A)** and distal **(B)** bone loss (mm) for platform switching (left) and platform matching (right) implants. Patients are categorized according to the Albrektsson Score into success (blue) and failure (red) groups

The Buser score indicated a high success rate across both groups, with 97.3% of implants considered successful (*p.* = 0.432). The Albrektsson score showed no significant difference between the groups, with 38.1% of implants in the platform switching group and 56.3% in the platform matching group classified as successful (*p.* = 0.272) (Fig. 1). The Attia score, a continuous measure of implant success, showed no significant difference between the platform switching (16.24 ± 1.04) and platform matching (13.71 ± 6.59) groups (*p.* = 0.794). Aesthetic outcomes, assessed by the Pink (Photograph) and Pink (Radiograph) scores, revealed no significant differences between the platform switching and platform matching groups (*p.* = 0.811 and *p.* = 1.000, respectively).

### Marginal bone loss

Mesial bone had a mean baseline level of 2.09 ± 1.22 mm and a mean current level of 2.57 ± 1.02 mm, with a mean total bone loss of 0.47 ± 1.10 mm (Table 3). No significant differences were found between the configuration groups in terms of baseline level or total bone loss. However, the current levels were significantly lower in the platform switching group (2.24 ± 0.74 mm) compared to the platform matching group (3.00 ± 1.19 mm, *p.* = 0.044). Distal bone levels, including baseline, current, and total bone loss, showed no significant differences between the groups (*p.* = 0.940 for baseline, *p.* = 0.728 for current, and *p.* = 0.774 for total bone loss).

**Table 3:** Marginal bone loss (MBL) of immediate implants inserted at University Hospital of Giessen and Marburg (UKGM) between 2002 and 2023, stratified by implant-abutment configuration (*n* = 37)

Side	Variable	Platform-switching (*n* = 21)	Platform-matching (*n* = 16)	Total (*n* = 37)	*p*.
**Mesial**	Baseline Bone Level	1.98 ± 1.16	2.24 ± 1.31	2.09 ± 1.22	0.404
	Current Bone Level	2.24 ± 0.74	3.00 ± 1.19	2.57 ± 1.02	**0.044**
	Total Bone Loss (Current – Baseline)	0.26 ± 1.12	0.75 ± 1.05	0.47 ± 1.10	0.476
**Distal**	Baseline Bone Level	1.74 ± 0.71	1.96 ± 1.40	1.84 ± 1.05	0.940
	Current Bone Level	2.42 ± 0.82	2.49 ± 1.17	2.45 ± 0.97	0.728
	Total Bone Loss (Current – Baseline)	0.68 ± 1.13	0.53 ± 1.21	0.62 ± 1.15	0.774

Mann-Whitney (*U*) test was used with a significance level (*p*.) < 0.05

No significant associations were found between total bone loss (TBL) and demographic characteristics such as sex (*p.* = 0.937 for mesial, *p.* = 0.649 for distal), age (*p.* = 0.914 for mesial, *p.* = 0.679 for distal), chronic illness (*p.* = 0.827 for mesial, *p.* = 0.639 for distal), medication use (*p.* = 0.572 for mesial, *p.* = 0.237 for distal), smoking status (*p.* = 0.433 for mesial, *p.* = 0.664 for distal), and toothbrushing habits (*p.* = 0.911 for mesial, *p.* = 0.570 for distal). These results indicate that these patient characteristics did not significantly impact bone loss outcomes (Table 4).

**Table 4:** Risk factors of total bone loss (TBL) of immediate implants inserted at University Hospital of Giessen and Marburg (UKGM) between 2002 and 2023 (*n* = 37)

Variable	Outcome	Mesial	*p.*	Distal	*p.*
**Sex**	Female	0.50 ± 0.86	0.937	0.75 ± 1.06	0.649
	Male	0.46 ± 1.23		0.55 ± 1.22	
**Age at Operation**	Correlation (*rho*)	-0.018	0.914	-0.070	0.679
**Chronic Illness**	No	0.41 ± 1.42	0.827	0.63 ± 1.09	0.639
	Yes	0.56 ± 0.67		0.64 ± 1.27	
**Medications**	No	0.46 ± 1.33	0.572	0.74 ± 1.13	0.237
	Yes	0.50 ± 0.70		0.44 ± 1.20	
**Smoking Status**	No	0.53 ± 1.18	0.433	0.58 ± 1.17	0.664
	Yes	0.29 ± 0.85		0.74 ± 1.14	
**Toothbrushing**	No	0.76	0.911	0.22	0.570
	Yes, twice a day	0.46 ± 1.15		0.66 ± 1.19	
	Yes, once a day	0.56 ± 0.35		0.15 ± 0.33	
**Jaw**	Upper	0.46 ± 1.13	0.814	0.62 ± 1.18	0.814
	Lower	0.69 ± 0.94		0.63 ± 1.05	
**Indication**	Trauma	0.41 ± 1.11	0.192	0.55 ± 1.16	0.588
	Explanation of previous implant	1.37 ± 0.86		1.26 ± 1.48	
	Hypodontia	-0.18		0.37	
	Root remnant	1.59		1.82	
**System**	BEGO	0.55 ± 1.15	0.277	0.62 ± 1.14	0.913
	XIVE	0.14 ± 0.85		0.66 ± 1.49	
	Straumann	-0.18		0.37	
**Implant Diameter**	3.75 *mm*	0.34 ± 0.41	0.423	0.12 ± 0.32	0.169
	4.10 *mm*	0.99 ± 1.04		0.98 ± 1.31	
	4.50 *mm*	0.24 ± 1.19		0.55 ± 1.17	
**Implant Length**	10 *mm*	-0.34	0.403	-0.67	0.748
	11.50 *mm*	0.53 ± 0.66		0.58 ± 0.85	
	12 *mm*	-0.18		0.37	
	13 *mm*	0.19 ± 1.28		0.62 ± 1.35	
	15 *mm*	0.86 ± 1.04		0.73 ± 1.14	
**Service Time**	Correlation (*rho*)	0.033	0.845	-0.056	0.744
**Superstructure**	VMK Single Crown	0.45 ± 1.16	0.886	0.60 ± 1.22	0.854
	Bridge	0.57		1.11	
	Overdenture	0.73 ± 0.54		0.60 ± 0.33	

Mann-Whitney (*U*) test and Kruskal-Wallis (*H*) test were used with a significance level (*p*.) < 0.05

Additional factors, including jaw location (*p.* = 0.814 for both mesial and distal), indication for implant placement (*p.* = 0.192 for mesial, *p.* = 0.588 for distal), implant system (*p.* = 0.277 for mesial, *p.* = 0.913 for distal), implant diameter (*p.* = 0.423 for mesial, *p.* = 0.169 for distal), implant length (*p.* = 0.403 for mesial, *p.* = 0.748 for distal), service time (*p.* = 0.845 for mesial, *p.* = 0.744 for distal), and superstructure type (*p.* = 0.886 for mesial, *p.* = 0.854 for distal), also showed no significant association with TBL.

### Regression models of marginal bone loss

Multiple linear regression analysis for mesial TBL demonstrated a good fit with an R² value of 0.521 (Table 5). The beta coefficient for the platform matching group was positive (β = 2.11, 95% CI: 0.11–4.11, *p.* = 0.039) compared to the platform switching group, indicating increased mesial bone loss with platform matching. This association remained significant after adjusting for service time. Males exhibited less mesial bone loss compared to females (β = -0.60, 95% CI: -1.74–0.55, *p.* = 0.286). Patients’ age at operation had a negligible effect (β = 0.00, 95% CI: -0.04–0.05, *p.* = 0.832). Chronic illness was associated with increased mesial TBL (β = 2.15, 95% CI: -0.12–4.42, *p.* = 0.062), while medication use reduced bone loss (β = -2.00, 95% CI: -4.33–0.33, *p.* = 0.088). Lower jaw had less bone loss, but this was not statistically significant (β = -0.54, 95% CI: -5.00–3.92, *p.* = 0.801). Additionally, a larger implant diameter tended to decrease mesial bone loss, although this association was not statistically significant (β = -2.08, 95% CI: -4.39–0.23, *p.* = 0.074).

**Table 5:** Multiple linear regression (MLR) of total bone loss (TBL) of immediate implants inserted at University Hospital of Giessen and Marburg (UKGM) between 2002 and 2023 (*n* = 37)

Predictor	Mesial TBL (R^2^ = 0.521)			Distal TBL (R^2^ = 0.440)		
	β (95% CI)	SE	*p.*	β (95% CI)	SE	*p.*
**Sex** (Male *vs.* Female)	-0.60 (-1.74–0.55)	0.54	0.286	-0.67 (-1.96–0.61)	0.61	0.285
**Age at Operation** (Continuous)	0.00 (-0.04–0.05)	0.02	0.832	-0.04 (-0.09–0.02)	0.03	0.186
**Chronic Illness** (Yes *vs.* No)	2.15 (-0.12–4.42)	1.08	0.062	2.38 (-0.17–4.94)	1.21	0.065
**Medications** (Yes *vs.* No)	-2.00 (-4.33–0.33)	1.11	0.088	-1.32 (-3.94–1.30)	1.24	0.303
**Smoking Status** (Yes *vs.* No)	-0.15 (-1.55–1.25)	0.66	0.824	-0.51 (-2.08–1.07)	0.75	0.506
**Toothbrushing** (Yes, twice daily *vs.* No)	1.85 (-5.65–9.36)	3.56	0.610	5.35 (-3.09–13.79)	4.00	0.199
**Toothbrushing** (Yes, once daily *vs.* No)	0.94 (-6.43–8.31)	3.49	0.791	3.28 (-5.01–11.57)	3.93	0.415
**Jaw** (Lower *vs.* Upper)	-0.54 (-5.00–3.92)	2.11	0.801	-0.11 (-5.13–4.90)	2.38	0.962
**Indication** (Prior Imp. Exp. *vs.* Trauma)	0.91 (-2.05–3.87)	1.40	0.525	2.56 (-0.77–5.89)	1.58	0.123
**Indication** (Hypodontia *vs.* Trauma)	1.23 (-3.09–5.54)	2.05	0.557	0.46 (-4.40–5.31)	2.30	0.845
**Indication** (Root remnant *vs.* Trauma)	1.97 (-2.54–6.48)	2.14	0.370	1.77 (-3.30–6.85)	2.40	0.471
**Implant Diameter** (Continuous)	-2.08 (-4.39–0.23)	1.09	0.074	-1.13 (-3.72–1.47)	1.23	0.371
**Implant Length** (Continuous)	0.33 (-0.14–0.80)	0.22	0.160	0.19 (-0.34–0.72)	0.25	0.460
**Service Time** (Continuous)	-0.23 (-0.50–0.04)	0.13	0.093	-0.21 (-0.51–0.10)	0.15	0.177
**Platform** (Matching *vs*. Switching)	2.11 (0.11–4.11)	0.95	**0.039**	0.88 (-1.37–3.12)	1.06	0.422
**Superstructure** (Bridge *vs.* VMK)	1.06 (-2.34–4.46)	1.61	0.519	2.29 (-1.54–6.11)	1.81	0.224
**Superstructure** (Overdenture *vs.* VMK)	0.09 (-3.05–3.23)	1.49	0.951	1.58 (-1.95–5.11)	1.67	0.359
**Implant System** (XIVE *vs*. BEGO)	-1.36 (-3.68–0.96)	1.10	0.234	-0.94 (-3.55–1.67)	1.24	0.459

The regression analysis for distal TBL also showed a good fit with an R² value of 0.440. The beta coefficient for the platform matching group was positive (β = 0.88, 95% CI: -1.37–3.12, *p.* = 0.422) compared to the platform switching group, indicating increased distal bone loss with platform matching. Males experienced less distal bone loss (β = -0.67, 95% CI: -1.96–0.61, *p.* = 0.285), while patients’ age at operation had minimal impact (β = -0.04, 95% CI: -0.09–0.02, *p.* = 0.186). Chronic illness was linked to increased distal TBL (β = 2.38, 95% CI: -0.17–4.94, *p.* = 0.065), whereas medication use was associated with decreased bone loss (β = -1.32, 95% CI: -3.94–1.30, *p.* = 0.303). Lower jaw also had less bone loss, but this was not statistically significant (β = -0.11, 95% CI: -5.13–4.90, *p.* = 0.962). Similarly, a larger implant diameter was associated with decreased distal bone loss, but this was not statistically significant (β = -1.13, 95% CI: -3.72–1.47, *p.* = 0.371).

## Discussion

The goal of this study was to compare two systems of immediately inserted dental implants, specifically focusing on the differences between platform-switching (PS) and platform-matching (PM) implant-abutment connections. There are very few studies on this topic, and the follow-up times and parameters of each study vary [3, 15]. The follow-up periods for studies focusing on immediately placed dental implants range from 12 months to 10 years [16–19]. In this study, the follow-up duration varied between 6 months and 23 years.

One of the most important factors for assessing implant success is marginal bone loss (MBL), which is a parameter reported in all studies on this topic. In a study by Pieri et al., MBL in the PS group was 0.2 ± 0.17 mm, and in the PM group, it was 0.51 ± 0.24 mm at a 12 months follow-up [16]. Crespi et al. reported no statistical difference between groups and with a MBL of 0.78 ± 0.49 mm for the PS group and 0.73 ± 0.52 mm for the control group after 24 months [19]. In a five-year observation by Slagter et al., MBL was reported as 0.71 ± 0.68 mm mesially and 0.71 ± 0.71 mm distally in PS implants, with the greatest occuring within the first month after implant placement [20].

Beschnidt et al., in a five-year follow-up of non-immediate implants placed in private practices, found no significant differences in mean bone level changes between PS and PM implants (− 0.32 ± 0.60 mm vs. −0.13 ± 0.29 mm), highlighting their comparable performance in clinical practice [21]. Canullo et al., in ten-year follow-up, found a significantly lower MBL in the PS group (0.18 ± 0.14 mm) compared to the control group (0.80 ± 0.40 mm) [17]. In the current study, MBL was 0.47 ± 1.13 mm for the PS group and 0.64 ± 1.13 mm for PM group. The difference between MBL for platform switching and platform matching was not statistically significant in this study, similar to the findings of Crespi et al. [19]. However, studies by Pieri and Canullo showed a significant advantage for PS implants (*p.* = 0.0004 and *p.* = 0.00108 respectively), with lower MBL values [16, 17]. Additionally, this study’s regression analysis indicated significantly lower mesial MPL in PS implants compared to PM implants, reinforcing previous research in favor of PS. These findings are consistent with a recently published systematic review by Vaghela et al., which concluded that the use of PS implants in immediate placement protocols can lead to a reduction in marginal bone loss compared to PM implants. However, the authors emphasized that these results should be interpreted with caution due to small sample sizes, substantial methodological variability in patients’ selection criteria and high risk of bias among the included studies [15]. Overall, while MBL values vary across studies, there is a slight trend favoring platform-switching implants in terms of bone preservation.

Additionally, the condition of the soft tissues around the implants should be evaluated. Probing depth (PD) is a useful measure for this purpose. When comparing the mesial, distal, vestibular and lingual PD values, no statistically significant differences were observed between the test and control groups. After averaging the four measured PD values, the mean PD can be compared with other studies. In this study, the mean PD for the PS group was 2.47 ± 0.13 mm, and for the PM group, it was 2.36 ± 0.15 mm. In comparison, Pieri’s study reported a PD of 2.58 ± 0.49 mm for the PS group and 2.71 ± 0.48 mm for the control group [16]. Overall, PD values did not show significant differences between studies or between study groups.

Inflammatory changes, implant stability and vertical bone loss were monitored in this study, along with the overall success of the implant using the implant score according to Buser and Albrektsson, as well as the newly published Attia score [22, 23]. The Attia score, in particular, offers a more nuanced evaluation by incorporating not only clinical and radiological parameters but also patient-centered outcomes, such as satisfaction with chewing function, speech ability, and aesthetic appearance. This multi-dimensional approach provides a more comprehensive understanding of implant success, considering both biological performance and patient quality of life. By combining these different scoring systems, this study aimed to provide a robust evaluation of implant outcomes over varying follow-up periods, enhancing our understanding of long-term implant success and patient satisfaction. However, future studies should assess the reproducibility and reliability of the Attia score in different populations, especially with longer-term follow-up data.

Although this retrospective study did not find a statistically significant difference between the PS and PM implant groups in the monitored factors, it provides a long-term picture of the functionality of both types of implants. Given that after 23 years both implant forms remain functional and no serious periodontal problems were observed, implantologists may be more inclined toward the PS form of the immediate implantation due to its potential advantages in preserving marginal bone levels and soft tissue stability.

As a limiting factor of this study, it is necessary to mention the smoking status of the patients. Smokers were included only in the PS group, representing 42.9% of the participants in this group. It is well known that smokers have a higher risk of implant failure and higher MBL values compared to non-smokers [24, 25]. In this study, only the test group, and not the control group, was affected by this factor. Pieri [16] reported that the number of smokers in their study was 7.5% (one participant in the test group and two in the control group). Other studies do not provide exact data on the proportion of smokers, making it difficult to compare and draw definitive conclusions based on this factor [9, 15]. In the context of this study, it can be stated that despite the negative impact of smoking, the PS group exhibited slightly better values than the PM group. To eliminate this bias in future studies, smokers should either be evenly distributed between study groups or excluded altogether to ensure a more balanced comparison.

Future research should focus on conducting prospective, randomized controlled trials with larger sample sizes to confirm the long-term advantages of platform switching implants, especially in smokers or patients with other risk factors that could influence implant success. Additionally, further studies should explore the biological mechanisms behind the differences in marginal bone loss between PS and PM implants, particularly in various clinical scenarios such as immediate versus delayed implantation, different bone qualities, and varying patient health conditions. Furthermore, integrating more advanced imaging techniques and biomarkers to track peri-implant tissue changes over time could help provide deeper insights into how platform switching affects the peri-implant environment. Finally, as patient satisfaction is becoming increasingly important in implantology, future studies should also explore how PS and PM implants influence long-term aesthetic outcomes and patient-reported quality of life, offering a more holistic understanding of implant success.

## Conclusions

This study compared the long-term performance of immediately placed platform switching (PS) and platform matching (PM) dental implants. Both systems showed stable clinical outcomes and high patient satisfaction over follow-up periods ranging from 6 months to 23 years, with no significant differences in probing depths, plaque index, or overall implant success. However, PS implants demonstrated significantly lower current mesial bone levels compared to PM implants, and regression analysis indicated greater mesial bone loss with PM implants. Despite both systems being viable options, platform switching may offer advantages in preserving peri-implant bone. Further prospective studies are needed to confirm these findings and explore the long-term benefits of PS implants, especially in relation to bone preservation in immediately placed dental implants.

## Electronic supplementary material

Below is the link to the electronic supplementary material.

Supplementary Material 1

Supplementary Material 2

## Data Availability

No datasets were generated or analysed during the current study.
