# Supplementary material for: The influence of platform switching and platform matching on marginal bone loss in immediately inserted dental implants: a retrospective clinical study
Source: Int J Implant Dent. 2025 Mar 4;11:16. doi: 10.1186/s40729-025-00604-y (PMC11880450; doi:10.1186/s40729-025-00604-y)
Supplement: Supplementary file 1 — Supplementary Material 1 [file 40729_2025_604_MOESM1_ESM.docx]

**Table 1.** Characteristics of Immediate Implants Inserted at University Hospital of Giessen and Marburg (UKGM) between 2002 and 2023, Stratified by Implant-abutment Configuration (*n* = 37)

| **Variable** | **Outcome** | **Platform-switching**  **(*n* = 21)** | **Platform-matching**  **(*n* = 16)** | **Total**  **(*n* = 37)** | ***p.*** |
| --- | --- | --- | --- | --- | --- |
| **Sex** | Female | 8 (38.1%) | 5 (31.3%) | 13 (35.1%) | 0.666 |
|  | Male | 13 (61.9%) | 11 (68.8%) | 24 (64.9%) |  |
| **Age at Operation** | Mean ± SD | 50.03 ± 20.66 | 41.62 ± 20.80 | 46.39 ± 20.86 | 0.175 |
| **Chronic Illness** | No | 11 (55%) | 8 (50%) | 19 (52.8%) | 0.765 |
|  | Yes | 9 (45%) | 8 (50%) | 17 (47.2%) |  |
| **Medications** | No | 15 (71.4%) | 7 (43.8%) | 22 (59.5%) | 0.089 |
|  | Yes | 6 (28.6%) | 9 (56.3%) | 15 (40.5%) |  |
| **Smoking Status** | No | 12 (57.1%) | 16 (100%) | 28 (75.7%) | **0.005** |
|  | Yes | 9 (42.9%) | 0 (0%) | 9 (24.3%) |  |
| **Toothbrushing** | No | 0 (0%) | 1 (6.3%) | 1 (2.7%) | 0.712 |
|  | Yes, twice a day | 20 (95.2%) | 14 (87.5%) | 34 (91.9%) |  |
|  | Yes, once a day | 1 (4.8%) | 1 (6.3%) | 2 (5.4%) |  |
| **Jaw** | Upper | 19 (90.5%) | 15 (93.8%) | 34 (91.9%) | 1.000 |
|  | Lower | 2 (9.5%) | 1 (6.3%) | 3 (8.1%) |  |
| **Indication** | Trauma | 20 (95.2%) | 13 (81.3%) | 33 (89.2%) | 0.117 |
|  | Prior implant explanation | 0 (0%) | 2 (12.5%) | 2 (5.4%) |  |
|  | Hypodontia | 0 (0%) | 1 (6.3%) | 1 (2.7%) |  |
|  | Root remnant | 1 (4.8%) | 0 (0%) | 1 (2.7%) |  |
| **System** | BEGO | 17 (81%) | 14 (87.5%) | 31 (83.8%) | 0.247 |
|  | XIVE | 4 (19%) | 1 (6.3%) | 5 (13.5%) |  |
|  | Straumann | 0 (0%) | 1 (6.3%) | 1 (2.7%) |  |
| **Implant Diameter** | 3.75 *mm* | 4 (19%) | 1 (6.3%) | 5 (13.5%) | 0.519 |
|  | 4.10 *mm* | 5 (23.8%) | 6 (37.5%) | 11 (29.7%) |  |
|  | 4.50 *mm* | 12 (57.1%) | 9 (56.3%) | 21 (56.8%) |  |
| **Implant Length** | 10 *mm* | 0 (0%) | 1 (6.3%) | 1 (2.7%) | **0.006** |
|  | 11.50 *mm* | 5 (23.8%) | 1 (6.3%) | 6 (16.2%) |  |
|  | 12 *mm* | 0 (0%) | 1 (6.3%) | 1 (2.7%) |  |
|  | 13 *mm* | 12 (57.1%) | 3 (18.8%) | 15 (40.5%) |  |
|  | 15 *mm* | 4 (19%) | 10 (62.5%) | 14 (37.8%) |  |
| **Service Time** | Mean ± SD | 3.18 ± 1.69 | 9.69 ± 4.09 | 5.99 ± 4.39 | **<0.001** |
| **Superstructure** | VMK Single Crown | 18 (85.7%) | 15 (93.8%) | 33 (89.2%) | 1.000 |
|  | Bridge | 1 (4.8%) | 0 (0%) | 1 (2.7%) |  |
|  | Overdenture | 2 (9.5%) | 1 (6.3%) | 3 (8.1%) |  |

Chi-squared (*χ^2^*) test, Fisher’s exact test, and Mann-Whitney (*U*) test was used with a significance level (*p*.) <0.05.

**Table 2.** Clinical Outcomes of Immediate Implants Inserted at University Hospital of Giessen and Marburg (UKGM) between 2002 and 2023, Stratified by Implant-abutment Configuration (*n* = 37)

| **Variable** | **Outcome** | **Platform-switching**  **(*n* = 21)** | **Platform-matching**  **(*n* = 16)** | **Total**  **(*n* = 37)** | ***p*.** |
| --- | --- | --- | --- | --- | --- |
| **Plaque Index** | 0 | 12 (57.1%) | 10 (62.5%) | 22 (59.5%) | 0.087 |
|  | 1 | 4 (19%) | 6 (37.5%) | 10 (27%) |  |
|  | 2 | 5 (23.8%) | 0 (0%) | 5 (13.5%) |  |
| **Mesial Probing** | 1 *mm* | 1 (5%) | 0 (0%) | 1 (2.8%) | 0.751 |
|  | 2 *mm* | 4 (20%) | 6 (37.5%) | 10 (27.8%) |  |
|  | 3 *mm* | 12 (60%) | 9 (56.3%) | 21 (58.3%) |  |
|  | 4 *mm* | 2 (10%) | 1 (6.3%) | 3 (8.3%) |  |
|  | 5 *mm* | 1 (5%) | 0 (0%) | 1 (2.8%) |  |
|  | Mean ± SD | 2.90 ± 0.85 | 2.69 ± 0.60 | 2.81 ± 0.75 | 0.440 |
| **Distal Probing** | 1 *mm* | 3 (15%) | 0 (0%) | 3 (8.3%) | 0.409 |
|  | 2 *mm* | 5 (25%) | 7 (43.8%) | 12 (33.3%) |  |
|  | 3 *mm* | 9 (45%) | 7 (43.8%) | 16 (44.4%) |  |
|  | 4 *mm* | 3 (15%) | 2 (12.5%) | 5 (13.9%) |  |
|  | Mean ± SD | 2.60 ± 0.94 | 2.69 ± 0.70 | 2.64 ± 0.83 | 0.937 |
| **Vestibular Probing** | 1 *mm* | 3 (15%) | 1 (6.3%) | 4 (11.1%) | 0.392 |
|  | 2 *mm* | 13 (65%) | 14 (87.5%) | 27 (75%) |  |
|  | 3 *mm* | 4 (20%) | 1 (6.3%) | 5 (13.9%) |  |
|  | Mean ± SD | 2.05 ± 0.61 | 2.00 ± 0.37 | 2.03 ± 0.51 | 0.814 |
| **Lingual Probing** | 1 *mm* | 2 (10.5%) | 2 (12.5%) | 4 (11.4%) | 0.825 |
|  | 2 *mm* | 10 (52.6%) | 11 (68.8%) | 21 (60%) |  |
|  | 3 *mm* | 6 (31.6%) | 3 (18.8%) | 9 (25.7%) |  |
|  | 4 *mm* | 1 (5.3%) | 0 (0%) | 1 (2.9%) |  |
|  | Mean ± SD | 2.32 ± 0.75 | 2.06 ± 0.57 | 2.20 ± 0.68 | 0.367 |
| **Complaint** | No | 15 (71.4%) | 11 (68.8%) | 26 (70.3%) | 1.000 |
|  | Yes | 6 (28.6%) | 5 (31.3%) | 11 (29.7%) |  |
| **Overall Satisfaction** | Very Good | 19 (90.5%) | 13 (81.3%) | 32 (86.5%) | 0.634 |
|  | Good | 2 (9.5%) | 3 (18.8%) | 5 (13.5%) |  |
| **Chewing** | Very Good | 17 (81%) | 13 (81.3%) | 30 (81.1%) | 1.000 |
|  | Good | 3 (14.3%) | 3 (18.8%) | 6 (16.2%) |  |
|  | Satisfactory | 1 (4.8%) | 0 (0%) | 1 (2.7%) |  |
| **Speech** | Very Good | 18 (85.7%) | 12 (75%) | 30 (81.1%) | 0.224 |
|  | Good | 3 (14.3%) | 1 (6.3%) | 4 (10.8%) |  |
|  | Satisfactory | 0 (0%) | 1 (6.3%) | 1 (2.7%) |  |
|  | Sufficient | 0 (0%) | 2 (12.5%) | 2 (5.4%) |  |
| **Buser Score** | Success | 21 (100%) | 15 (93.8%) | 36 (97.3%) | 0.432 |
|  | Failure | 0 (0%) | 1 (6.3%) | 1 (2.7%) |  |
| **Albrektsson Score** | Success | 8 (38.1%) | 9 (56.3%) | 17 (45.9%) | 0.272 |
|  | Failure | 13 (61.9%) | 7 (43.8%) | 20 (54.1%) |  |
| **Attia Score** | Mean ± SD | 16.24 ± 1.04 | 13.71 ± 6.59 | 15.11 ± 4.58 | 0.794 |
| **Pink Score (Photograph)** | Mean ± SD | 11.37 ± 3.08 | 11.13 ± 2.53 | 11.26 ± 2.81 | 0.811 |
| **Pink Score (Radiograph)** | Mean ± SD | 11.79 ± 1.55 | 11.80 ± 1.37 | 11.79 ± 1.45 | 1.000 |

Chi-squared (*χ^2^*) test, Fisher’s exact test, and Mann-Whitney (*U*) test was used with a significance level (*p*.) <0.05.

**Table 3.** Marginal Bone Loss (MBL) of Immediate Implants Inserted at University Hospital of Giessen and Marburg (UKGM) between 2002 and 2023, Stratified by Implant-abutment Configuration (*n* = 37)

| **Side** | **Variable** | **Platform-switching**  **(*n* = 21)** | **Platform-matching**  **(*n* = 16)** | **Total**  **(*n* = 37)** | ***p*.** |
| --- | --- | --- | --- | --- | --- |
| **Mesial** | Baseline Bone Level | 1.98 ± 1.16 | 2.24 ± 1.31 | 2.09 ± 1.22 | 0.404 |
|  | Current Bone Level | 2.24 ± 0.74 | 3.00 ± 1.19 | 2.57 ± 1.02 | **0.044** |
|  | Total Bone Loss (Current – Baseline) | 0.26 ± 1.12 | 0.75 ± 1.05 | 0.47 ± 1.10 | 0.476 |
| **Distal** | Baseline Bone Level | 1.74 ± 0.71 | 1.96 ± 1.40 | 1.84 ± 1.05 | 0.940 |
|  | Current Bone Level | 2.42 ± 0.82 | 2.49 ± 1.17 | 2.45 ± 0.97 | 0.728 |
|  | Total Bone Loss (Current – Baseline) | 0.68 ± 1.13 | 0.53 ± 1.21 | 0.62 ± 1.15 | 0.774 |

Mann-Whitney (*U*) test was used with a significance level (*p*.) <0.05.

**Table 4.** Risk Factors of Total Bone Loss (TBL) of Immediate Implants Inserted at University Hospital of Giessen and Marburg (UKGM) between 2002 and 2023 (*n* = 37)

| **Variable** | **Outcome** | **Mesial** | ***p.*** | **Distal** | ***p.*** |
| --- | --- | --- | --- | --- | --- |
| **Sex** | Female | 0.50 ± 0.86 | 0.937 | 0.75 ± 1.06 | 0.649 |
|  | Male | 0.46 ± 1.23 |  | 0.55 ± 1.22 |  |
| **Age at Operation** | Correlation (*rho*) | -0.018 | 0.914 | -0.070 | 0.679 |
| **Chronic Illness** | No | 0.41 ± 1.42 | 0.827 | 0.63 ± 1.09 | 0.639 |
|  | Yes | 0.56 ± 0.67 |  | 0.64 ± 1.27 |  |
| **Medications** | No | 0.46 ± 1.33 | 0.572 | 0.74 ± 1.13 | 0.237 |
|  | Yes | 0.50 ± 0.70 |  | 0.44 ± 1.20 |  |
| **Smoking Status** | No | 0.53 ± 1.18 | 0.433 | 0.58 ± 1.17 | 0.664 |
|  | Yes | 0.29 ± 0.85 |  | 0.74 ± 1.14 |  |
| **Toothbrushing** | No | 0.76 | 0.911 | 0.22 | 0.570 |
|  | Yes, twice a day | 0.46 ± 1.15 |  | 0.66 ± 1.19 |  |
|  | Yes, once a day | 0.56 ± 0.35 |  | 0.15 ± 0.33 |  |
| **Jaw** | Upper | 0.46 ± 1.13 | 0.814 | 0.62 ± 1.18 | 0.814 |
|  | Lower | 0.69 ± 0.94 |  | 0.63 ± 1.05 |  |
| **Indication** | Trauma | 0.41 ± 1.11 | 0.192 | 0.55 ± 1.16 | 0.588 |
|  | Explanation of previous implant | 1.37 ± 0.86 |  | 1.26 ± 1.48 |  |
|  | Hypodontia | -0.18 |  | 0.37 |  |
|  | Root remnant | 1.59 |  | 1.82 |  |
| **System** | BEGO | 0.55 ± 1.15 | 0.277 | 0.62 ± 1.14 | 0.913 |
|  | XIVE | 0.14 ± 0.85 |  | 0.66 ± 1.49 |  |
|  | Straumann | -0.18 |  | 0.37 |  |
| **Implant Diameter** | 3.75 *mm* | 0.34 ± 0.41 | 0.423 | 0.12 ± 0.32 | 0.169 |
|  | 4.10 *mm* | 0.99 ± 1.04 |  | 0.98 ± 1.31 |  |
|  | 4.50 *mm* | 0.24 ± 1.19 |  | 0.55 ± 1.17 |  |
| **Implant Length** | 10 *mm* | -0.34 | 0.403 | -0.67 | 0.748 |
|  | 11.50 *mm* | 0.53 ± 0.66 |  | 0.58 ± 0.85 |  |
|  | 12 *mm* | -0.18 |  | 0.37 |  |
|  | 13 *mm* | 0.19 ± 1.28 |  | 0.62 ± 1.35 |  |
|  | 15 *mm* | 0.86 ± 1.04 |  | 0.73 ± 1.14 |  |
| **Service Time** | Correlation (*rho*) | 0.033 | 0.845 | -0.056 | 0.744 |
| **Superstructure** | VMK Single Crown | 0.45 ± 1.16 | 0.886 | 0.60 ± 1.22 | 0.854 |
|  | Bridge | 0.57 |  | 1.11 |  |
|  | Overdenture | 0.73 ± 0.54 |  | 0.60 ± 0.33 |  |

Mann-Whitney (*U*) test and Kruskal-Wallis (*H*) test were used with a significance level (*p*.) <0.05.

**Table 5.** Multiple Linear Regression (MLR) of Total Bone Loss (TBL) of Immediate Implants Inserted at University Hospital of Giessen and Marburg (UKGM) between 2002 and 2023 (*n* = 37)

| **Predictor** | **Mesial TBL (R^2^ = 0.521)** | | | **Distal TBL (R^2^ = 0.440)** | | |
| --- | --- | --- | --- | --- | --- | --- |
|  | **β (95% CI)** | **SE** | ***p.*** | **β (95% CI)** | **SE** | ***p.*** |
| **Sex** (Male *vs.* Female) | -0.60 (-1.74 – 0.55) | 0.54 | 0.286 | -0.67 (-1.96 – 0.61) | 0.61 | 0.285 |
| **Age at Operation** (Continuous) | 0.00 (-0.04 – 0.05) | 0.02 | 0.832 | -0.04 (-0.09 – 0.02) | 0.03 | 0.186 |
| **Chronic Illness** (Yes *vs.* No) | 2.15 (-0.12 – 4.42) | 1.08 | 0.062 | 2.38 (-0.17 – 4.94) | 1.21 | 0.065 |
| **Medications** (Yes *vs.* No) | -2.00 (-4.33 – 0.33) | 1.11 | 0.088 | -1.32 (-3.94 – 1.30) | 1.24 | 0.303 |
| **Smoking Status** (Yes *vs.* No) | -0.15 (-1.55 – 1.25) | 0.66 | 0.824 | -0.51 (-2.08 – 1.07) | 0.75 | 0.506 |
| **Toothbrushing** (Yes, twice daily *vs.* No) | 1.85 (-5.65 – 9.36) | 3.56 | 0.610 | 5.35 (-3.09 – 13.79) | 4.00 | 0.199 |
| **Toothbrushing** (Yes, once daily *vs.* No) | 0.94 (-6.43 – 8.31) | 3.49 | 0.791 | 3.28 (-5.01 – 11.57) | 3.93 | 0.415 |
| **Jaw** (Lower *vs.* Upper) | -0.54 (-5.00 – 3.92) | 2.11 | 0.801 | -0.11 (-5.13 – 4.90) | 2.38 | 0.962 |
| **Indication** (Prior Imp. Exp. *vs.* Trauma) | 0.91 (-2.05 – 3.87) | 1.40 | 0.525 | 2.56 (-0.77 – 5.89) | 1.58 | 0.123 |
| **Indication** (Hypodontia *vs.* Trauma) | 1.23 (-3.09 – 5.54) | 2.05 | 0.557 | 0.46 (-4.40 – 5.31) | 2.30 | 0.845 |
| **Indication** (Root remnant *vs.* Trauma) | 1.97 (-2.54 – 6.48) | 2.14 | 0.370 | 1.77 (-3.30 – 6.85) | 2.40 | 0.471 |
| **Implant Diameter** (Continuous) | -2.08 (-4.39 – 0.23) | 1.09 | 0.074 | -1.13 (-3.72 – 1.47) | 1.23 | 0.371 |
| **Implant Length** (Continuous) | 0.33 (-0.14 – 0.80) | 0.22 | 0.160 | 0.19 (-0.34 – 0.72) | 0.25 | 0.460 |
| **Service Time** (Continuous) | -0.23 (-0.50 – 0.04) | 0.13 | 0.093 | -0.21 (-0.51 – 0.10) | 0.15 | 0.177 |
| **Platform** (Matching *vs*. Switching) | 2.11 (0.11 – 4.11) | 0.95 | **0.039** | 0.88 (-1.37 – 3.12) | 1.06 | 0.422 |
| **Superstructure** (Bridge *vs.* VMK) | 1.06 (-2.34 – 4.46) | 1.61 | 0.519 | 2.29 (-1.54 – 6.11) | 1.81 | 0.224 |
| **Superstructure** (Overdenture *vs.* VMK) | 0.09 (-3.05 – 3.23) | 1.49 | 0.951 | 1.58 (-1.95 – 5.11) | 1.67 | 0.359 |
| **Implant System** (XIVE *vs*. BEGO) | -1.36 (-3.68 – 0.96) | 1.10 | 0.234 | -0.94 (-3.55 – 1.67) | 1.24 | 0.459 |
