# Supplementary material for: The influence of platform switching and platform matching on marginal bone loss in immediately inserted dental implants: a retrospective clinical study
Source: Int J Implant Dent. 2025 Mar 4;11:16. doi: 10.1186/s40729-025-00604-y (PMC11880450; doi:10.1186/s40729-025-00604-y)
Supplement: Supplementary file 2 — Supplementary Material 2 [file 40729_2025_604_MOESM2_ESM.pdf]

The Influence of Platform Switching and Platform Matching on Marginal Bone Loss in Immediately Inserted Dental Implants: A Retrospective Clinical Study

Effects of platform switching (PS) and platform matching (PM) on marginal bone loss (MBL) and clinical parameters in immediately inserted dental implants

Methods

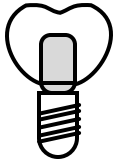

PS
n = 21

vs.

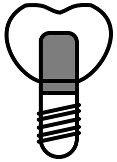

PM
n = 16

Retrospective design

- Clinical outcomes
- X-ray analysis
  MBL [mm] on mesial (m) and distal (d) side
- Regression models of total bone loss

Results

- No differences in probing depths, plaque index, or overall implant success
- MBL PS: 0.26 (m) and 0.68 (d); MBL PM: 0.75 (m) and 0.53 (d)
- Lower current mesial bone levels in PS group (*p.* = 0.044)
- PM implants were associated with greater mesial bone loss (*p.* = 0.039)

Despite both systems being viable options, platform switching may offer advantages in preserving peri-implant bone in immediately inserted dental implants.
